# Dressing-induced hemodynamic instability in patients with heart failure: Implications for nursing care

**DOI:** 10.1371/journal.pone.0351501

**Published:** 2026-06-11

**Authors:** Tae Watanabe, Kimiko Tagawa, Yu Kimura, Toshiro Kitagawa, Kiyokazu Sekikawa, Yukiko Nakano, Sanae Oriyama

**Affiliations:** 1 Doctoral Program in Biomedical and Health Sciences, Hiroshima University, Hiroshima, Japan; 2 Department of Nursing, Faculty of Nursing, Yasuda Women’s University, Hiroshima, Japan; 3 Department of Health Informatics, Graduate School of Biomedical and Health Sciences, Hiroshima University, Hiroshima, Japan; 4 Department of Nursing, Faculty of Human Health Sciences, Shunan Public University, Yamaguchi, Japan; 5 Department of Cardiovascular Medicine, Graduate School of Biomedical Sciences, Hiroshima University, Hiroshima, Japan; 6 Department of Biofunction Analysis and Control Science, Graduate School of Biomedical Sciences, Hiroshima University, Hiroshima, Japan; 7 Department of Basic Nursing, Division of Nursing, Graduate School of Biomedical and Health Sciences, Hiroshima University, Hiroshima, Japan; Showa University: Showa Daigaku, JAPAN

## Abstract

**Background:**

Heart failure (HF) restricts activities of daily living, impacting prognosis and quality of life. Dressing requires sustained upper-limb movements, postural transitions, and fine-motor tasks that may impose cardiovascular and autonomic demands, potentially informing on physiological tolerance during daily activities.

**Objective:**

This observational study compared hemodynamic, autonomic, subjective, and upper-limb sensor count (ULSC) responses during dressing across HF clinical courses to characterize recovery dynamics during daily activities and identify nursing support requirements.

**Methods:**

We compared healthy controls (HCs; n = 15), *de novo* HF (NO-HF; n = 12), and recurrent HF (R-HF; n = 12) groups. Heart rate (HR), systolic blood pressure (SBP), peripheral oxygen saturation, HR variability, Borg scale score, ULSC, dressing duration, and HR recovery (HRR) were measured at rest, during dressing, and after 20 min of recovery. Linear mixed-effects models tested group, time, and interaction. Pairwise comparisons were performed via Bonferroni adjustment.

**Results:**

HR increased during and immediately post-dressing across groups, with NO-HF exhibiting higher HR than R-HF. HRR varied among groups (HC vs. NO-HF: P = 0.108; HC vs. R-HF: P < 0.0001; NO-HF vs. R-HF: P = 0.026). SBP displayed a group effect (P = 0.034). Peak HR responses tended to occur immediately post-dressing in HC and NO-HF, whereas R-HF descriptively showed a delayed peak around 5 min. No significant group × time interaction was observed. ULSC showed no group differences after dressing time and body size adjustments. Patients with HF had higher Borg scores than did HCs (NO-HF, P < 0.001; R-HF, P = 0.028).

**Conclusions:**

Despite being low-intensity, dressing was associated with measurable physiological and subjective HF responses. R-HF trended toward slower HR recovery; NO-HF demonstrated consistently higher HR levels and perceived exertion. Monitoring recovery responses and supporting pacing and symptom self-monitoring during daily activities may be important in HF nursing care.

## Introduction

Heart failure (HF) is a progressive clinical syndrome characterized by impaired cardiac function and autonomic dysregulation, leading to reduced exercise tolerance and limitations in activities of daily living (ADLs) [1–3]. In patients with HF, post-activity regulation of heart rate (HR) and blood pressure is often delayed, causing unstable hemodynamic recovery [4,5], which is associated with rehospitalization, poor quality of life, and adverse outcomes [4–7]. Research has focused predominantly on cardiovascular and autonomic responses during exercise testing or structured rehabilitation [8–10]. However, physiological responses to low-intensity ADLs, such as dressing, particularly during the task and early recovery period (0–20 min), remain underexplored. Dressing is an essential ADL involving sustained upper-limb activity, postural transitions, and fine motor control that can impose a disproportionate cardiovascular/autonomic load and elicit symptoms, such as dyspnea and fatigue, in patients with HF [11,12]. Given the established association between ADL impairment and diminished quality of life [7,13,14], characterizing physiological stress during routine activities is clinically meaningful.

ADL studies involving dressing have compared oxygen uptake and symptoms between patients with HF and healthy controls (HCs) [11,12]. However, within-HF stratification (*de novo* HF [NO-HF] vs. recurrent HF [R-HF]; HF with preserved ejection fraction vs. HF with reduced ejection fraction) at the physiological level remains limited. Functional limitations in HF with preserved ejection fraction have been described; however, direct ADL physiology comparisons are rare [10,15]. Moreover, study designs that concurrently capture hemodynamics (HR, systolic blood pressure [SBP], oxygen saturation [SpO₂]), autonomic indices (HR variability [HRV]), subjective symptom burden (Borg), and behavior (upper-limb sensor count [ULSC]) within a single protocol that continuously evaluates responses from task to recovery are uncommon [7,11,12]. NO-HF and R-HF are different phenotypes with distinct characteristics and outcomes [16,17]. Chronic or recurrent disease is associated with progressive structural remodeling, neurohormonal activation, and autonomic imbalance [18,19]. Consequently, even low-intensity ADL responses may differ across groups. However, the correlation among compensatory behavior during ADLs, cardiovascular/autonomic responses, and subjective symptom burden remains poorly understood.

We aimed to characterize simultaneous hemodynamic, autonomic, subjective symptom burden (Borg), and behavioral (ULSC) responses to a standardized dressing task across HCs and patients with either NO-HF or R-HF using a time-resolved, longitudinal comparison. We sought to elucidate how recovery delay/instability relates to compensatory behavior, thereby informing nursing practice (ADL safety assessment, self-pacing, rest guidance, and patient education). We hypothesized that compared with NO-HF and HC, R-HF would exhibit delayed HR recovery (HRR) and blunted HR responses, SBP would display unstable fluctuations during early recovery, and HF groups would exhibit persistent autonomic imbalance, greater compensatory upper-limb activity, and higher subjective symptom burden.

## Materials and methods

### Study design and participants

Participants were recruited between April 2024 and July 2025. Eligible individuals included outpatients aged 60–79 years who had been treated as inpatients for HF, who were at least 6 months post-discharge, as well as age-matched HCs. The ≥ 6 months post-discharge criterion was established to ensure clinical stability and minimize acute phase hemodynamic and autonomic parameter fluctuations. Previous studies have reported that physiological and functional recovery within the 3–6 months post-hospitalization for HF varies substantially depending on disease severity, adherence, and comorbidities [20–22]. Therefore, the ≥ 6-month threshold aligned with previous research involving stable outpatients to improve the interpretability and comparability of results.

The HF group was subdivided according to hospitalization history: NO-HF (one hospitalization) and R-HF (two or more hospitalizations). This grouping strategy was selected to reflect cumulative disease burden and recurrent decompensation in clinically stable outpatients with HF, rather than to define physiological HF subtypes. As the primary objective of this study was to characterize recovery dynamics during activities of daily living rather than to compare ejection fraction-based pathophysiological phenotypes, hospitalization frequency was used as a clinically meaningful indicator of disease trajectory and readmission risk in patients with HF. To complement this classification, the New York Heart Association (NYHA) functional class and left ventricular ejection fraction (LVEF) suggested that participants with R-HF had greater functional impairment than did those with NO-HF.

The exclusion criteria were irregular R–R intervals, implanted pacemakers, history of cardiac catheter ablation, dependence in executing ADLs, or a history of dementia. Patients who were ADL-dependent or cognitively impaired were excluded to ensure safe execution of the dressing task and reliable acquisition of physiological data, because these conditions may interfere with task performance and autonomic measurement accuracy.

### Sample size estimation

A priori power analysis was conducted using G*Power version 3.1.9.7 (Heinrich-Heine-Universität Düsseldorf, Düsseldorf, Germany) [23] using the “analysis of variance “ANOVA”: Repeated measures, within–between interaction” option. The parameters were effect size f = 0.25 (moderate), two-sided α = 0.05, power (1 − β) = 0.95, three groups, and seven repeated measurements (rest, dressing, immediately after, 5, 10, 15, and 20 min). The analysis indicated a minimum sample size of 33 patients (approximately 11 patients per group). To account for attrition and potential sphericity violations, we targeted ≥12 participants per group and eventually enrolled 39 participants (male, n = 24; mean age 70.4 ± 6.1 years) (HCs, n = 15; NO-HF, n = 12; R-HF, n = 12), thereby exceeding the required number.

### Rationale for the assumed effect size

An effect size of f = 0.25 was selected as a moderate effect according to Cohen’s convention for ANOVA designs [24,25], which aligned with methodological recommendations emphasizing that researchers should explicitly justify assumed effect sizes and acknowledge uncertainty, particularly when prior empirical estimates are lacking [26].

Although no previous research has examined dressing-specific cardiovascular responses in HF, ADL- and submaximal activity-based studies in HF populations have reported medium-range physiological differences (e.g., oxygen uptake, HR, or symptom responses) between patients with HF and controls [12,27,28]. Therefore, a moderate effect was assumed as a methodologically conservative estimate to balance feasibility and statistical power. We acknowledge that, if the true effect was smaller, this study may be underpowered to detect subtle between-group differences.

### Study protocol

**Fig 1:**
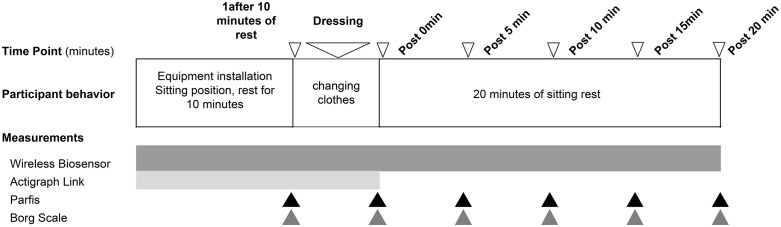
Study protocol.

The study protocol is illustrated in Fig 1. The dressing procedure involved putting on tops and bottoms as underwear and then a round-neck long-sleeved top, long pants with elastic waistbands, and socks, and then removing and putting on the same type of top, bottoms, and socks while still wearing the underwear. Measurements were taken at the following time points: rest (after 10 min of rest), dressing, post 0 min (immediately after dressing), post 5 min, post 10 min, post 15 min, and post 20 min.

Measurements were conducted at participants’ homes to observe natural dressing behavior. Assessments were conducted 2–3 h after a meal to minimize the influence of postprandial hypotension, which is common among older adults and patients with HF [29–31]. To reduce potential confounding effects, participants were instructed to avoid caffeine and alcohol after 9:00 p.m. the previous night and to refrain from vigorous physical activity within 3 h before assessment, while maintaining usual medication schedules on the day of assessment. Measurements were conducted between 10:00 a.m. and 3:00 p.m. Room temperature was maintained between 20 and 26 °C, corresponding to the thermal comfort range for sedentary activities defined by the American Society of Heating, Refrigerating and Air-Conditioning Engineers Standard 55 [32]. Although home-based measurements increase ecological validity, environmental factors, such as room temperature, lighting, and ambient noise, cannot be fully controlled, which may influence cardiovascular and autonomic responses.

The dressing task simulated typical daily activities. The participants wore familiar clothing that was pre-specified to standardize the type and sequence of garments. Participants removed a pullover long-sleeved shirt, and elastic waist long pants, worn over their underwear (undershirt and underpants), and socks, then dressed a mannequin with a folded set of identical replacement garments in the same order. All garments were lightweight and non-restrictive to ensure ecological validity and safety.

Each participant underwent a single measurement, with each session lasting for approximately 40 min. Because intra- and inter-day variability were not assessed, future studies should include repeated measurements or standardized environmental conditions to enhance reproducibility and internal validity.

### Measurements

#### Hemodynamics and autonomic function.

Hemodynamic indices included HR, SBP, percutaneous arterial SpO₂, and HRR. Autonomic function was evaluated using HRV indices: coefficient of variation of R–R intervals (CvRR), low-frequency power (LF: 0.04–0.15 Hz), high-frequency power (HF: 0.15–0.40 Hz), LF/HF ratio, and normalized low-frequency power (LF-NU).

Continuous monitoring of the HR and autonomic indices was performed using a wireless biosensor (RF-ECG2, GMS Co. Ltd., Tokyo, Japan). R–R interval time-series data were analyzed in real time using MemCalc/Bonaly Light (GMS), recording the HR and activity every 2 s. Measurements were conducted at seven time points: rest (10 min), during dressing, immediately after dressing, and at 5, 10, 15, and 20 min post-task. SBP and SpO₂ were measured using a portable device (Palfis WB-100, Nippon Seimitsu Sokki, Japan) at six time points: at rest, immediately after dressing, and at 5, 10, 15, and 20 min post-task.

HRR was defined as the time required for the HR to return to within ±1 bpm of the baseline resting value and remain stable for 10 s after task completion. HRR reflects parasympathetic reactivation after exercise and has been shown to be a clinically important prognostic indicator in patients with HF [4,5,33].

LF and HF reflect sympathetic and parasympathetic activities, respectively, and the LF/HF ratio is widely used as an index of autonomic balance. These frequency-domain measures have been reported to be useful for evaluating autonomic dysfunction and predicting the prognosis of patients with HF [34–37].

#### Activity characteristics.

Activity characteristics were evaluated using ULSC and dressing duration. The ULSC during dressing was measured using an accelerometer-based device (ActiGraph Link GT9X; ActiJapan, Japan) worn on the left wrist. Acceleration, gyroscopy, and geomagnetic sensor data were continuously recorded from rest until the end of the dressing task for approximately 15 min, with analysis performed every 2 s. The three-axis activity counts (axis 1: vertical; axis 2: horizontal; and axis 3: lateral) were summed to obtain the total ULSC.

To account for differences in dressing duration, the total ULSC score was normalized to the task time (counts/s) to enable between-group comparisons. For group comparisons, ULSC was analyzed as time-normalized counts (counts/s) relative to body size (body mass index [BMI]). Dressing duration was determined using MemCalc/Bonaly Light (GMS, Japan); the start and end of dressing were marked, and the elapsed time between the markers was calculated in seconds.

#### Subjective symptom burden.

The subjective symptom burden associated with ADLs was assessed using the Borg scale and specific symptom reports. Participants were requested to rate their symptoms at six time points: at rest, immediately after dressing, and 5, 10, 15, and 20 min after dressing.

### Statistical analysis

Results are expressed as mean ± standard deviation (SD), with 10-min seated rest as baseline. A two-way linear mixed model analysis of variance was performed with group (R-HF, NO-HF, and HC; three groups) and time (seven time points: rest, during dressing, immediately after dressing, 5, 10, 15, and 20 min) as factors. Post hoc comparisons with Bonferroni corrections were conducted relative to baseline values. ULSC was compared between groups as time-normalized counts with BMI considered in the analysis.

For HRR, dressing duration, and ULSC, a one-way linear mixed model analysis of variance was performed. Statistical analyses were conducted using IBM SPSS Statistics version 20.0 J for Windows (IBM Corp., Armonk, NY, USA) and R software version 4.4.0 (R Foundation for Statistical Computing, Vienna, Austria). A level of 5% was considered statistically significant.

### Ethical considerations

This study was approved by the Epidemiological Research Ethics Committee of Hiroshima University (approval number: E2023-0264). Written informed consent was obtained from all participants before enrollment. The study protocol complied with the Declaration of Helsinki.

### Safety of home-based measurements

Given that older patients with HF are at risk of hemodynamic instability, we established a system to communicate self-checks and predefined stop criteria (e.g., chest pain, dyspnea, and dizziness), immediate cessation and contact with the primary physician or emergency services upon symptom onset, immediate cessation upon device malfunction, and documentation and reporting of adverse events, per Institutional Review Board policy (none occurred).

## Results

### Participant characteristics

The baseline characteristics of the study participants (mean age: 70.4 ± 6.1 years) are presented in Table 1. The study included 39 individuals (R-HF, n = 12; NO-HF, n = 12; and HC, n = 15). There were no significant differences among the three groups regarding sex, age, or BMI (Table 1). To improve clinical characterization of the HF population, LVEF-based HF phenotypes (HF with reduced ejection fraction, HF with mildly reduced ejection fraction, and HF with preserved ejection fraction) are additionally summarized in Table 1.

**Table 1:** Characteristics of participants.

	Total (n = 39)	Recurrent HF (n = 12)	New-onset HF (n = 12)	Healthy controls (n = 15)	P-value
**Sex: Male, n**	24	10	8	6	0.17
**Age (years)**	70 (6)	72 (6)	69 (7)	71 (6)	0.61
**BMI (kg/m²)**	23.1 (4.2)	22.0 (3.3)	23.4 (2.5)	22.5 (2.2)	0.44
**Left ventricular ejection fraction (%)**		38.3 (14.4)	48.2 (13.4)	－	－
**HFrEF (<40%)**	9	5	4	－	－
**HFmrEF (40–49%)**	6	4	2	－	－
**HFpEF (≥50%)**	9	3	6	－	－
**NYHA class I/II/III/IV**		0/10/2/0	7/5/0/0	0/0/0/0	－
Comorbidities (n, multiple counts allowed)					
Ischemic heart disease	16	7	9	0	－
Valvular disease	7	6	1	0	－
Cardiomyopathy	3	2	1	0	－
Others	1	1	0	0	－
Hypertension	20	6	6	8	－
History of cardiac surgery	18	9	9	0	－
Diabetes mellitus	9	3	5	1	－
Dyslipidemia	6	3	3	0	－
Renal failure	5	4	1	0	－
Malignancy	5	2	3	0	－

Values are presented as mean ± standard deviation or number.

LVEF values are shown for patients with HF only.

HF phenotypes were classified according to LVEF as follows: HFrEF, < 40%; HFmrEF, 40–49%; and HFpEF, ≥ 50%.

BMI, body mass index; HF, heart failure; HFrEF, heart failure with reduced ejection fraction; HFmrEF, heart failure with mildly reduced ejection fraction; HFpEF, heart failure with preserved ejection fraction; LVEF, left ventricular ejection fraction; NYHA, New York Heart Association functional classification.

### Baseline values before dressing

At rest, no significant differences were observed among the three groups in any of the physiological variables, thereby ensuring the validity of subsequent comparisons (Table 2).

**Table 2:** Baseline characteristics at rest (sitting for 10 min).

	HC	NO-HF	R-HF	P-value
**HR** (bpm)	69.7 (7.8)	74.3 (14.1)	66.6 (8.6)	0.95
**SBP** (mmHg)	127.8 (13.6)	119.8 (19.1)	117.3 (14.2)	0.34
**SpO**_**2**_ (％)	98.1 (0.5)	97.3 (1.4)	98.0 (0.7)	0.47
**CvRR** (％)	2.9 (1.0)	2.9 (1.9)	2.3 (1.8)	0.37
**LF** (ms^2^)	178.6 (162.7)	124.5 (148.1)	209.9 (347.4)	0.32
**HF** (ms^2^)	89.7 (103.9)	179.4 (455.0)	67.8 (108.0)	0.21
**LF/HF**	5.1 (7.3)	4.4 (3.7)	3.3 (2.9)	0.35
**LF-NU** (％)	61.3 (25.6)	70.8 (21.7)	64.3 (25.1)	0.34

Values are presented as mean (standard deviation).

HR, heart rate; CvRR, coefficient of variation of R–R intervals; LF, low-frequency power; HF, high-frequency power; LF/HF, ratio of low- to high-frequency power; NU, normalized unit; SBP, systolic blood pressure; SpO₂, peripheral oxygen saturation.

P-values were calculated for group comparisons; P < 0.05 was considered statistically significant.

### Changes in physiological parameters during dressing

Statistical analyses are summarized in Tables 3–5. Among the main effects, significant group differences were observed in HR, SBP, SpO₂, CvRR, LF, and Borg scale scores (Table 4). Significant time effects were observed for HR, CvRR, and Borg scale scores (Table 5). No significant group × time interactions were identified. For HF, LF/HF ratio, and LF-NU, neither group nor time effects were significant, indicating that these indices were not influenced by the presence of heart disease or measurement time points.

**Table 3:** Linear mixed model analysis: physiological parameters, subjective outcome measure.

Outcome measures	Group		TP		Group * TP	
	F DF	P-value	F DF	P-value	F DF	P-value
**HR** (bpm)	7.55 (252)	**<0.001**	13.91 (252)	**<0.001**	0.15 (252)	1.000
**SBP** (mmHg)	3.59 (212)	**0.029**	0.71 (212)	0.613	0.45 (212)	0.928
**SpO**_**2**_ (％)	7.90 (206)	**<0.001**	0.49 (206)	0.786	0.17 (206)	0.998
**CvRR** (％)	4.62 (252)	**<0.001**	5.041 (252)	**<0.001**	0.75 (252)	0.143
**LF** (ms^2^)	3.05 (252)	**0.049**	0.39 (252)	0.888	0.26 (252)	0.995
**HF** (ms^2^)	0.12 (252)	0.883	0.16 (252)	0.988	0.80 (252)	0.646
**LF/HF**	1.69 (252)	0.186	0.67 (252)	0.676	1.02 (252)	0.427
**LF-NU** (％)	2.04 (252)	0.132	0.96 (252)	0.454	0.87 (252)	0.58
**Borg scale** (score)	8.07 (216)	**<0.001**	5.16 (216)	**<0.001**	1.49 (216)	0.145

F, variation between sample means; DF, degrees of freedom; TP, time point.

Bold values indicate statistically significant differences (P < 0.05).

HR, heart rate; CvRR, coefficient of variation of R–R intervals; LF, low-frequency power; HF, high-frequency power; LF/HF, ratio of low- to high-frequency power; NU, normalized unit; SBP, systolic blood pressure; SpO₂, peripheral oxygen saturation.

**Table 4:** Linear mixed model analysis: multiple comparisons of variables with significant group differences among physiological parameters.

Outcome measures	HC:NO-HF	HC:R-HF	NO-HF:R-HF
	P-value	P-value	P-value
**HR** (bpm)	0.092	0.168	**<0.001**
**SBP** (mmHg)	0.202	**0.034**	1.000
**SpO**_**2**_ (％)	**<0.001**	1.000	0.006
**CvRR** (％)	**<0.001**	0.064	1.000
**LF** (ms^2^)	**0.043**	0.686	0.699
**Borg scale** (score)	**<0.001**	**0.028**	0.688

HR, heart rate; CvRR, coefficient of variation of R–R intervals; LF, low-frequency power; HF, high-frequency power; LF/HF, ratio of low- to high-frequency power; SBP, systolic blood pressure; SpO₂, peripheral oxygen saturation.

P-values were calculated using Bonferroni correction for multiple comparisons. P < 0.05 was considered statistically significant. Bold values indicate statistical significance.

**Table 5:** Linear mixed model analysis: multiple comparisons of variables with significant time-point differences among physiological parameters.

Outcome measures	Dressing	Immediately after	5 min	10 min	15 min	20 min
	P-value	P-value	P-value	P-value	P-value	P-value
**HR** (bpm)	**<0.001**	**<0.001**	1.000	1.000	1.000	1.000
**CvRR** (％)	0.052	1.000	1.000	1.000	1.000	1.000
**Borg scale** (score)	**―**	**<0.001**	0.426	1.000	1.000	1.000

HR, heart rate; CvRR, coefficient of variation of R–R intervals; Borg scale, subjective rating of perceived exertion.

P-values were calculated using Bonferroni correction for multiple comparisons. P < 0.05 was considered statistically significant. Bold values indicate statistical significance.

### Hemodynamics

#### HR.

**Fig 2:**
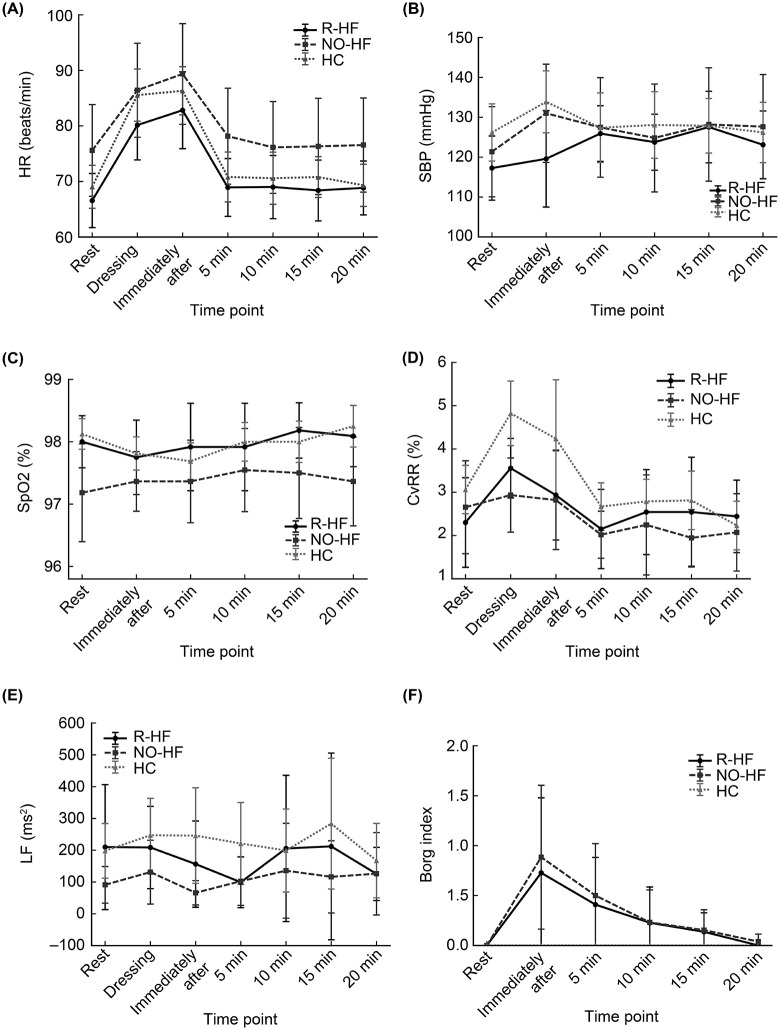
Group time courses of physiological and subjective responses around a dressing task. **(A)** Heart rate (HR), (B) systolic blood pressure (SBP), (C) oxygen saturation (SpO₂), (D) coefficient of variation of R–R intervals (CvRR), (E) low-frequency HRV power (LF), and **(F)** Borg scale (0–10). Lines depict group means with 95% confidence intervals (CIs); time points are Rest, Dressing, Immediately after, 5, 10, 15, and 20 min. For SBP, SpO₂, and Borg, values were not recorded during dressing; lines connect Rest to Immediately after. In panel **(F)**, lower 95% CIs are truncated at 0 to respect the bounded scale; healthy controls reported 0 at all time points (no symptoms). Group coding: healthy controls (HC, light gray dotted), *de novo* HF (NO-HF, dark gray dashed), and recurrent HF (R-HF, black solid). Sample sizes: HC n = 15, NO-HF n = 12, R-HF n = 12. Statistical inferences are reported in the Results and Tables.

Linear mixed model analysis revealed the significant main effects of both group and time on HR, although no significant interaction was observed (Table 3). Across all groups, the HR significantly increased during and immediately following dressing (both P < 0.001) and subsequently returned to baseline over time (Table 5, Fig 2A). In the group comparison, HR was significantly higher in NO-HF than in R-HF (P < 0.001; Table 4). The mean peak HR values ranged from 66.6 to 86.2 bpm across all participants. The differences between resting and peak HR were 16.2, 14.3, and 17.0 bpm in the R-HF, NO-HF, and HC groups, respectively (Fig 2A).

#### HRR.

**Fig 3:**
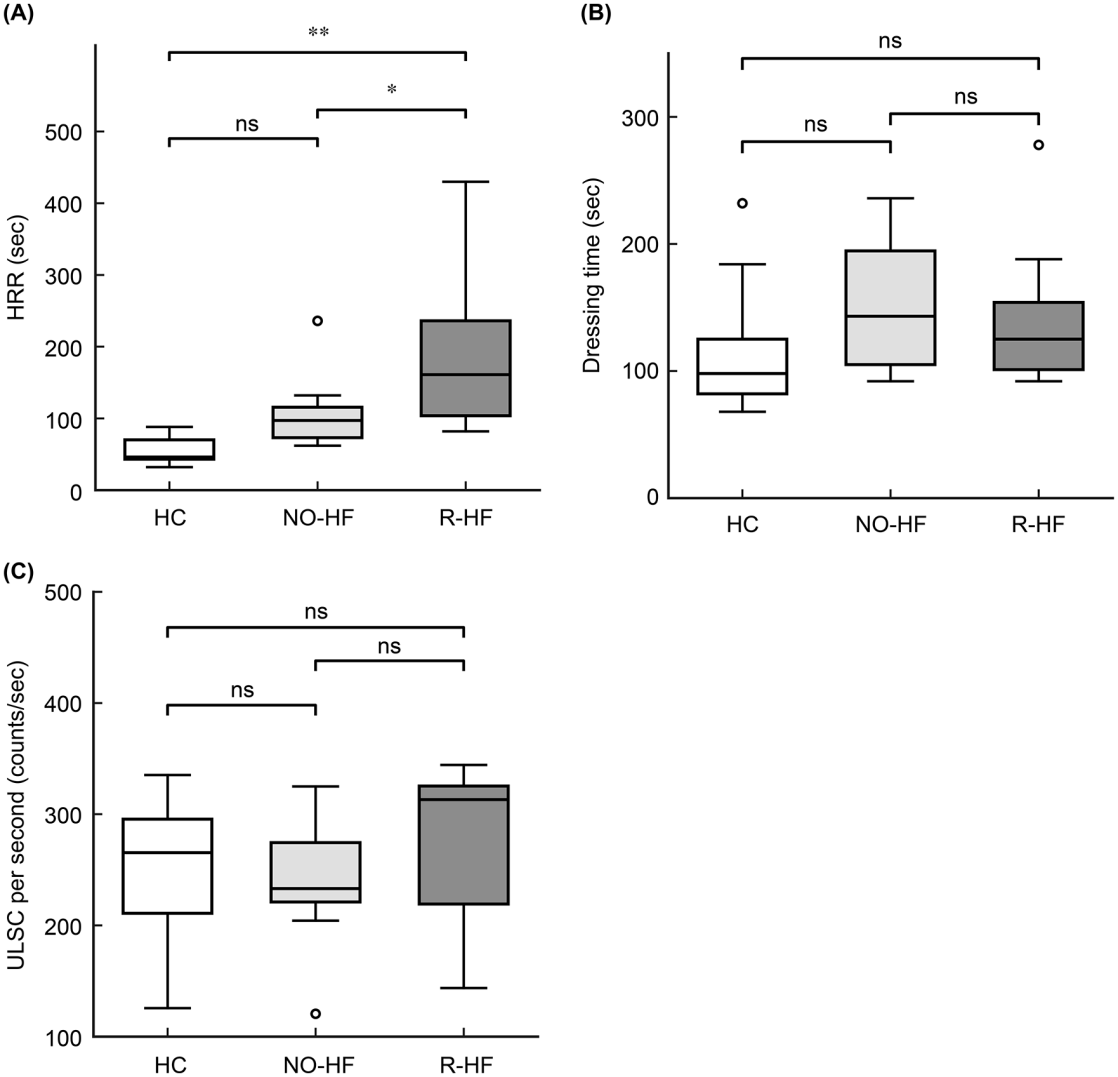
Group comparisons of HRR, dressing time, and ULSC normalized by time (ULSC/s). **(A)** HRR (s) by group (boxplot), **(B)** Boxes indicate the interquartile range (IQR) with median lines; whiskers represent 1.5 × IQR; open circles denote outliers. Fill scheme: HC = light gray, NO-HF = dark gray, R-HF = white (black outline). In panels **(A)**–(C), horizontal bars depict pairwise group comparisons with Bonferroni-adjusted significance shown by asterisks (*P < 0.05; **P < 0.01; ns = not significant). Time (s) by group (boxplot), **(C)** ULSC per second (counts/s) by group (boxplot). Sample sizes. **(A)** HRR: HC n = 15, NO-HF n = 12, R-HF n = 12. **(B)** Dressing time: HC n = 15, NO-HF n = 12, R-HF n = 12. **(C)** ULSC/s: HC n = 15, NO-HF n = 12, R-HF n = 10 (cases with missing ULSC excluded). Abbreviations: HRR, heart rate recovery; ULSC, upper-limb sensor counts; HC, healthy control; NO-HF, *de novo* heart failure; R-HF, recurrent heart failure. Note: The primary analysis for ULSC used a generalized linear model with a log link and an offset of log (dressing time), with body mass index as a covariate; pairwise comparisons were Bonferroni-adjusted. Panel (C) provides a descriptive visualization of time-normalized ULSC; inferential results are based on the model.

The mean (SD) HRR was 56.0 (17.2) s in HC, 114.7 (60.4) s in NO-HF, and 193.5 (108.5) s in R-HF. Group comparisons using linear modeling revealed that the HRR was significantly longer in R-HF than in HC (P < 0.0001) and NO-HF (P = 0.026) (Table 6, Fig 3A). These findings demonstrate that delayed HRR occurred in patients with HF after a daily dressing task, with this impairment being most pronounced in the R-HF group.

**Table 6:** Heart rate recovery, dressing duration, and upper-limb activity counts among groups.

	R-HF	NO-HF	HC
**HRR** (s)	193.5 (108.5)	114.7(60.4)	56.0 (17.2)
**Dressing duration** (s)	138.3 (54.2)	150.5 (51.4)	111.5 (47.4)
**ULSC** (count)	7,190 (7,161)	34,807 (9,874)	26,313 (7,098)

Values are presented as mean (standard deviation).

HRR: heart rate recovery; ULSC: upper-limb sensor counts; R-HF: recurrent heart failure; NO-HF: new-onset heart failure; HC: healthy controls.

### SBP

For SBP, the group effect was significant, whereas the time and group × time interaction effects were not (Table 3). Post hoc analysis revealed that HCs had significantly higher SBP than that in R-HF (P = 0.034), while no significant differences were observed between the HC and NO-HF groups or between the NO-HF and R-HF groups (Table 4). Regarding temporal changes, SBP tended to peak immediately after dressing in the HC and NO-HF groups, whereas in the R-HF group, the peak tended to occur 5 min post-task. Subsequently, SBP in HCs promptly returned to baseline, whereas NO-HF and R-HF descriptively exhibited a small secondary SBP rise at approximately 15 min; however, this pattern should be interpreted cautiously because no significant group × time interaction was identified (Fig 2B).

### SpO₂

SpO₂ showed a significant main effect of group but no significant effects of time or group × time interaction (Table 3). Post hoc comparisons revealed that HCs had a significantly higher SpO₂ than that in NO-HF (P < 0.001), whereas no significant differences were found between HC and R-HF or between NO-HF and R-HF (Table 4). Importantly, all participants maintained SpO₂ levels above 95%, and no significant temporal changes were observed during or after dressing (Table 5; Fig 2C).

### HRV

#### CvRR.

Linear mixed model analysis revealed a significant main effect of group on CvRR, whereas no significant interaction was observed (Table 3). In the group comparison, NO-HF showed significantly lower CvRR than that of HCs (P < 0.001), whereas no significant differences were observed between the HC and R-HF groups or between the NO-HF and R-HF groups (Table 4). Across all groups, CvRR peaked during dressing and returned to baseline by 5 min post-task. The mean differences between the resting and peak values were 1.3% for R-HF, 0.2% for NO-HF, and 1.9% for HC, suggesting a blunted CvRR response to dressing in the NO-HF group (Fig 2D).

#### LF, HF, LF-NU, and LF/HF.

For LF, only the main effect of group was significant. Post hoc analysis revealed a significant difference between the HC and NO-HF groups (P = 0.043), but no significant differences were detected across time points (Tables 4 and 5; Fig 2E). Conversely, HF, LF-NU, and LF/HF showed no significant main effects or interactions (Tables 3–5; S1a–c Fig), indicating that these indices were not influenced by group or time.

### Activity characteristics

#### Dressing duration.

The mean (SD) dressing duration was 138.3 (54.2) s in R-HF, 150.5 (51.4) s in NO-HF, and 111.5 (47.4) s in HC (Table 6). Group comparisons revealed no statistically significant between-group differences (Fig 3B). These findings indicate that patients with HF performed dressing tasks within a timeframe comparable to that of HC.

#### ULSC.

The ULSC was analyzed as time-normalized counts (counts/s) with respect to body size (BMI). No significant between-group differences were observed (HC vs. NO-HF, P = 0.909; HC vs. R-HF, P = 0.636; R-HF vs. NO-HF, P = 0.569; Bonferroni-adjusted comparisons were not significant). Dressing duration did not differ among the groups, and time-normalized ULSC did not differ across groups, indicating that apparent group differences in unadjusted totals likely reflect exposure time or body size rather than compensatory overuse (Table 6, Fig 3C; S1d Fig).

### Subjective symptom burden (Borg scale)

The Borg scale scores showed significant main effects for both group and time but no significant interaction (Table 3). Post hoc comparisons revealed that Borg scores were higher in HF than in HC (NO-HF vs. HC: P < 0.001; R-HF vs. HC: P = 0.028), with that of NO-HF being the highest (Tables 4 and 5; Fig 2F). Compared with those at rest, the Borg scores increased significantly immediately after dressing (P < 0.001) and then gradually decreased, approaching asymptomatic levels from 5 min onward. Reported symptoms include shortness of breath, dyspnea, and chest discomfort. These results indicate that, unlike HCs, who consistently reported a score of 0, both HF groups, particularly the NO-HF group, experienced a transient increase in subjective symptom burden during dressing, which subsided with subsequent rest.

## Discussion

This study showed that even a low-intensity dressing task revealed exploratory differences in the quality of recovery (speed and stability) among patients with HF and that NO-HF and R-HF may manifest distinct vulnerability patterns. Using a single protocol, we directly compared R-HF, NO-HF, and HC while concurrently assessing the cardiovascular (HR/SBP), autonomic (HRV), behavioral (ULSC), and subjective (Borg) domains. Below, we appraise each a priori hypothesis against the data and integrate the mechanisms with those of previous literature.

### H1-1: Hierarchy of HRR impairment (R-HF > NO-HF > HC)

A prespecified hierarchy was observed: HRR was the slowest in R-HF, intermediate in NO-HF, and fastest in HC (overall P < 0.001; pairwise R-HF vs. HC P < 0.0001). This pattern is consistent with impaired vagal reactivation and delayed sympathovagal withdrawal after activity, which correlate with adverse outcomes when HRR is blunted [38]. In this context, the present ranking may reflect a gradient of autonomic recovery reserves with recurrent decompensation accompanied by deeper impairment. Nevertheless, because group × time interaction effects were not statistically significant, these findings should be interpreted cautiously and considered exploratory observations warranting confirmation in larger cohorts.

In the present study, HF groups were classified according to clinical course (new-onset vs. recurrent HF) to reflect cumulative disease burden and recurrent decompensation in daily clinical practice. Accordingly, the primary aim was to characterize recovery dynamics during activities of daily living rather than to compare ejection fraction-based pathophysiological HF phenotypes.

Notably, LVEF-based HF phenotypes were distributed across both HF groups, suggesting that the observed recovery patterns were not attributable to a single EF category alone.

### H1-2: Blunted HR response in R-HF and high-level persistence in NO-HF are partially supported

HR increased immediately post-dressing across groups but remained consistently higher in the NO-HF group from rest onward (high-level persistence), whereas the R-HF group exhibited a smaller response amplitude (blunting). The apparent time-to-peak at approximately 5 min in the R-HF group is descriptive (no significant interaction), yet it may be compatible with patterns described in the chronotropic incompetence: an inadequate HR increase for metabolic demand and delayed recovery [39]. Chronotropic incompetence is frequent in HF and is associated with reduced functional capacity; our “blunted-and-delayed” R-HF profile and delayed HRR may reflect physiology consistent with this mechanism. Nonetheless, because medication use and clinical severity markers were unavailable in this study, this interpretation should be regarded as exploratory. Particularly, the potential influence of pharmacological HR modulation cannot be excluded. In contrast, NO-HF may be characterized by elevated set-point/persistent tachycardia rather than response failure, suggesting potentially distinct recovery response patterns within HF groups [40].

### H1-3: Instability of SBP recovery-strongly partially supported

A significant group main effect of SBP was detected (P = 0.034). Graphically, the HF group exhibited a small re-rise at approximately 15 min, and the R-HF group displayed a tendency toward a later SBP peak (~5 min). Nonetheless, these temporal features were descriptive rather than statistically confirmed interactions. Mechanistically, upper-limb tasks can provoke greater HR and BP responses than lower-limb tasks at comparable metabolic loads, due to smaller active muscle mass and higher peripheral resistance [40–42]. During early recovery, arterial baroreflex function is reset rather than “switched off,” operating at a higher pressure set-point and potentially demonstrating transient instability while reflexes re-equilibrate [43,44]. Together, these may account for the 10–20-min window wherein we observed circulatory vulnerability (re-elevation/instability).

### H2: Sustained autonomic imbalance/upper limb activity/perceived exertion is partially supported

Among the autonomic indices, LF demonstrated a group effect (HC ≠ NO-HF, P = 0.043), whereas HF, LF/HF, and LF-NU were non-significant. However, interpreting LF/HF or LF-NU as a simple “sympathetic/parasympathetic balance” remains controversial, particularly in short epochs and low-intensity tasks, because these metrics are heavily influenced by vagal modulation and analytic choices and exhibit inconsistent responses during exercise/recovery [45,46]. Accordingly, the HRV findings in this study should be interpreted cautiously and considered supportive rather than definitive indicators of autonomic imbalance.

Behaviorally, ULSC standardized by dressing duration (counts/s) and adjusted for covariates yielded no group differences; Borg was higher in HF, being the highest in NO-HF, mirroring the high-HR persistent profile. Synthesizing H1 and H2, these findings may suggest two complementary recovery response patterns relevant to bedside care: NO-HF = high HR level + higher perceived load and R-HF = blunted HR rise + delayed HRR. ULSC should therefore be interpreted primarily as an upper-limb activity volume indicator rather than a direct marker of compensatory motor strategies.

### Clinical/nursing implications

For R-HF, monitoring was emphasized through approximately 15 min of recovery, including a check for late SBP rise as a simple, teachable pattern. For NO-HF, pacing strategies (insert rest, avoid continuous upper-limb sequences), and self-monitoring of HR/effort are recommended. Across HF phenotypes, beyond single-point SpO₂/HR, documenting the recovery profile (speed, variability, and any re-elevation), may provide a more sensitive functional indicator following ADLs. This aligns with rehabilitation evidence suggesting that multidomain early functional training enhances performance in vulnerable populations with HF [47].

### Limitations

This study has some limitations. First, the group definition relied solely on the readmission count without adjunctive biomarkers and detailed HF phenotype characterization, which may have diluted subgroup specificity. Although LVEF-based HF phenotypes were additionally summarized, biomarkers reflecting HF severity, including B-type natriuretic peptide and N-terminal pro-B-type natriuretic peptide, were not collected. Therefore, detailed characterization of disease severity and physiological heterogeneity across HF groups remains limited. Second, excluding ADL-dependent patients and those with dementia limited the generalizability to high-need HF subgroups. Third, non-standardized environmental variability (room temperature/illumination/noise/home setting) and the single-session design precluded the assessment of day-to-day variability, thereby affecting reliability. Fourth, detailed medication data, including β-blockers, angiotensin-converting enzyme (ACE) inhibitors/angiotensin II receptor blockers/angiotensin receptor–neprilysin inhibitors, mineralocorticoid receptor antagonists, and diuretics, were not systematically collected. These therapies may have influenced HR, HRR, SBP, and autonomic responses; therefore, the findings should be interpreted cautiously. Fifth, medication timing (β-blockers, diuretics, and ACE inhibitors) was imperfectly controlled and could alter acute responses. Sixth, all participants wore the device on the left wrist, and hand dominance was not recorded, which may have influenced the ULSC due to laterality bias. Future protocols should correct for side dominance. Seventh, the assumed effect size (f = 0.25) lacked strong empirical grounding and should be viewed as a methodological limitation.

For interpretation, SBP re-rise and the R-HF ~ 5-min HR peak are descriptive (exploratory) features; these patterns should therefore be interpreted cautiously because group × time interaction effects were not statistically significant. Moreover, short-epoch HRV during low-intensity nonstationary segments is not a simple surrogate for sympathovagal balance [8,9]. Looking ahead, areas under the curve, time-to-peak, and recovery time constants should be pre-registered; spline/time-series models, 30–60-min monitoring, and concurrent respiration/actigraphy with medication-timing sensitivity analyses could more robustly test the chain from “quality of recovery after ADL” → “function and outcomes.”

## Conclusions

Even a low-intensity dressing task revealed exploratory differences in recovery quality among groups: HRR ranked as R-HF > NO-HF > HC. NO-HF tended to show persistently higher HR levels with higher Borg ratings, while R-HF descriptively showed a blunted HR rise with delayed HR recovery. In addition, NO-HF and R-HF descriptively demonstrated a small SBP re-rise at approximately 15 min of recovery. Given that no significant group × time interactions were identified, these findings should be cautiously interpreted as exploratory recovery patterns requiring confirmation in larger studies. Clinically, we recommend monitoring through 15 min post-ADL with a routine SBP re-elevation check, prioritizing pacing and self-monitoring education in NO-HF, and documenting recovery profiles rather than single time-point vitals.

## Supporting information

S1 Fig Changes in autonomic responses and upper-limb activity during and after dressing. (a) High-frequency (HF) power, (b) low-frequency to HF ratio (LF/HF), and (c) LF power in normalized units (LF-NU) are presented across time points: Rest, dressing, immediately after, and 5–20 min after dressing. These indices reflect changes in parasympathetic and sympathetic modulation during recovery. (d) The relationship between upper-limb sensor counts (ULSC, total counts) and dressing time (s) showing that longer dressing duration was associated with greater total limb movement across groups. Data are shown as mean ± SD. R-HF, recurrent heart failure; NO-HF, de novo heart failure; HC, healthy controls; SD, standard deviation.
(DOCX)
