# Supplementary material for: Dressing-induced hemodynamic instability in patients with heart failure: Implications for nursing care
Source: PLoS One. 2026 Jun 11;21(6):e0351501. doi: 10.1371/journal.pone.0351501 (PMC13257999; doi:10.1371/journal.pone.0351501)
Supplement: S1 Fig — (a) High-frequency (HF) power, (b) low-frequency to HF ratio (LF/HF), and (c) LF power in normalized units (LF-NU) are presented across time points: Rest, dressing, immediately after, and 5–20 min after dressing. These indices reflect changes in parasympathetic and sympathetic modulation during recovery. (d) The relationship between upper-limb sensor counts (ULSC, total counts) and dressing time (s) showing that longer dressing duration was associated with greater total limb movement across groups. Data are shown as mean ± SD. R-HF, recurrent heart failure; NO-HF, de novo heart failure; HC, healthy controls; SD, standard deviation. (DOCX) [file pone.0351501.s001.docx]

**Supporting Information**


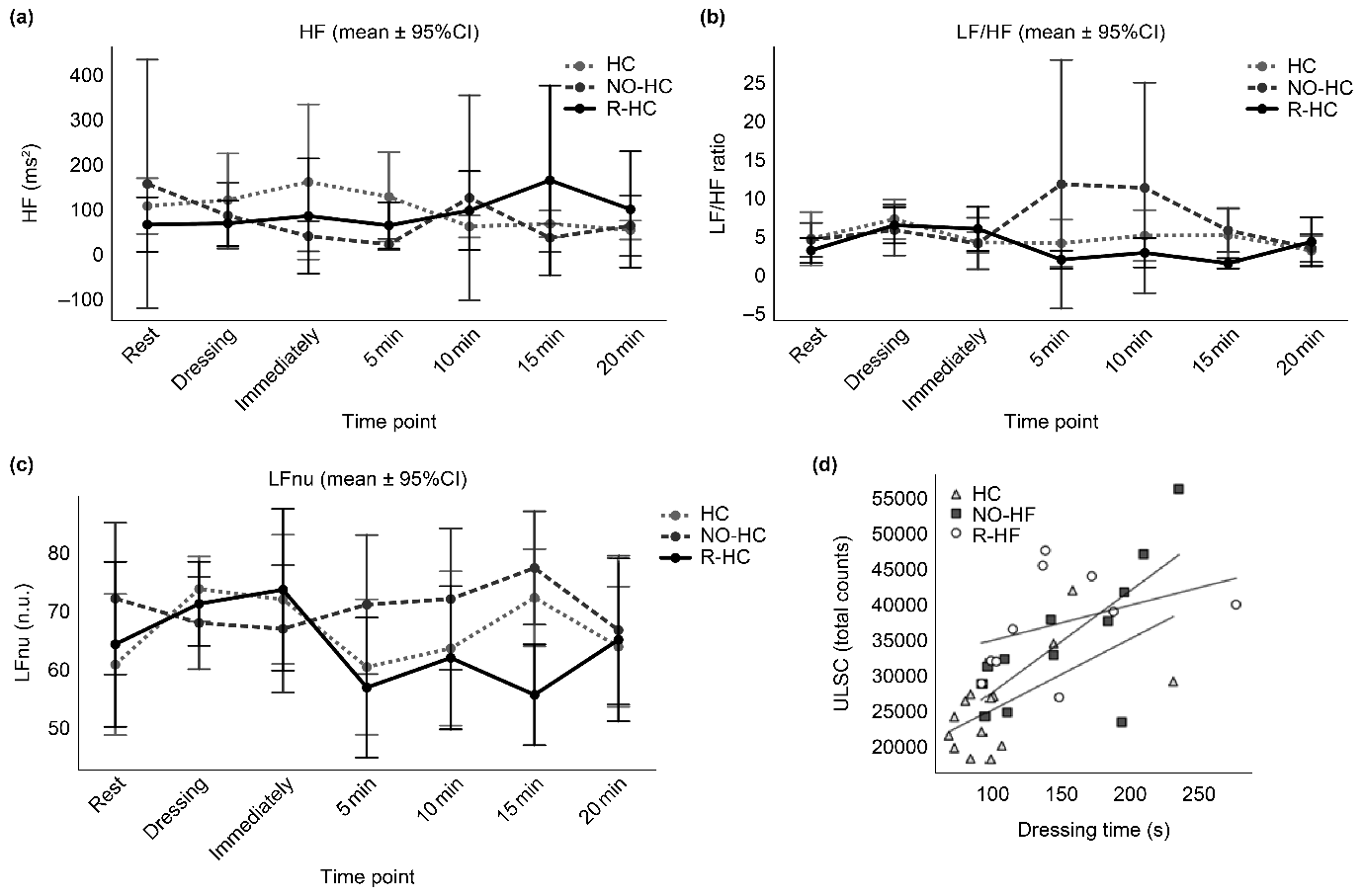


**S1 Fig. Changes in autonomic responses and upper-limb activity during and after dressing.**

(a) High-frequency (HF) power, (b) low-frequency to HF ratio (LF/HF), and (c) LF power in normalized units (LF-NU) are presented across time points: Rest, dressing, immediately after, and 5–20 min after dressing. These indices reflect changes in parasympathetic and sympathetic modulation during recovery. (d) The relationship between upper-limb sensor counts (ULSC, total counts) and dressing time (s) showing that longer dressing duration was associated with greater total limb movement across groups. Data are shown as mean ± SD.

R-HF, recurrent heart failure; NO-HF, de novo heart failure; HC, healthy controls.
