# Supplementary material for: Dressing-induced hemodynamic instability in patients with heart failure: Implications for nursing care
Source: PLoS One. 2026 Jun 11;21(6):e0351501. doi: 10.1371/journal.pone.0351501 (PMC13257999; doi:10.1371/journal.pone.0351501)
Supplement: Attachment — Submitted filename: Response to reviewers.docx [file pone.0351501.s002.docx]

**Response to Reviewers**

Manuscript ID: **PONE-D-25-63210**

Title: Dressing-induced hemodynamic instability in patients with heart failure: Implications for nursing care

We sincerely thank the Academic Editor and the reviewers for their careful evaluation of our manuscript and for their insightful and constructive comments. We greatly appreciate the time and expertise devoted to reviewing our work. We have carefully considered all comments and revised the manuscript accordingly. All revisions are indicated using track changes in the revised manuscript.

**Response to Academic Editor**

Comment 1
Please ensure that the manuscript complies with PLOS ONE style requirements.

Response
Thank you for your helpful comment. The manuscript has been carefully reviewed and revised to ensure full compliance with the PLOS ONE style and formatting requirements. Specifically, we have verified the manuscript structure, section formatting, references, and file preparation to ensure adherence to the journal guidelines.

Manuscript change
Formatting and style corrections have been applied throughout the manuscript.

Comment 2

Please revise the Funding Statement to comply with journal format and include the sentence: “There was no additional external funding received for this study.”

Response
We appreciate this comment. Accordingly, the Funding Statement has been revised to ensure full compliance with the journal’s requirements. As requested, the sentence “There was no additional external funding received for this study.” has been added to clarify that this study did not receive any additional external funding beyond the support already acknowledged.

Manuscript change
The Funding Statement has been revised.

Comment 3

Please revise the Data Availability Statement to clarify ethical or legal restrictions on sharing the dataset.

Response
Thank you for this important comment. The dataset used in this study contains potentially identifiable human participant information collected during clinical assessments. Therefore, public sharing of the dataset is restricted in accordance with the ethics approval obtained from the Ethics Committee of Hiroshima University. To ensure transparency regarding these restrictions, we have revised the Data Availability Statement to clearly explain the ethical limitations governing data sharing.

Manuscript change
The Data Availability Statement has been revised to clarify the ethical restrictions imposed by the Ethics Committee of Hiroshima University.

Comment 4
Please provide non-author contact information for data access requests.

Response
We appreciate this important suggestion. To comply with the PLOS ONE data sharing policy while protecting participant confidentiality, we have revised the Data Availability Statement to include a non-author institutional contact responsible for ethical oversight and data access requests. The institutional contact provided is the Ethics Committee of Hiroshima University. Researchers who meet the criteria for access to confidential data may contact the committee directly to request access.

Institutional contact for data access requests:
Ethics Committee of Hiroshima University
E-mail: [iryo-sinsa@office.hiroshima-u.ac.jp](mailto:iryo-sinsa@office.hiroshima-u.ac.jp)
TEL: +81-82-257-5947
TEL: +81-82-257-1946
URL: <https://ethics.hiroshima-u.ac.jp/%e5%a7%94%e5%93%a1%e4%bc%9a%e4%b8%80%e8%a6%a7-%e7%9b%b8%e8%ab%87%e7%aa%93%e5%8f%a3/>

Manuscript change
Institutional contact information has been added to the Data Availability Statement.

**Reviewer 1**

Comment 1
The sample size is relatively small.

Response
Thank you for this critical comment. We agree that the sample size was relatively modest and may have limited the statistical power to detect subtle differences between groups. Therefore, we have explicitly acknowledged this limitation in the revised manuscript and clarified that the findings should be interpreted with caution. Future studies with larger cohorts are warranted to confirm our findings.

Manuscript change
A statement regarding the limited sample size has been added to the Limitations section.

Comment 2
Group × time interaction effects were not statistically significant.

Response
We appreciate the reviewer’s careful observation. We agree that the absence of statistically significant group × time interaction effects limits the strength of conclusions regarding temporal differences between groups. Therefore, we have revised the manuscript to ensure that temporal patterns are described as descriptive or exploratory observations rather than statistically confirmed interaction effects.

Manuscript change
Relevant sentences in the Discussion section have been revised to emphasize cautious interpretation of temporal patterns.

Comment 3
Please clarify the definition of heart rate recovery (HRR).

Response
Thank you for your helpful suggestion. We have clarified the operational definition of HRR in the Methods section. In this study, HRR was defined as the time required for heart rate to return to within ±1 bpm of the resting baseline value and remain stable for at least 10 s after completion of the dressing task. This definition was selected to capture recovery dynamics following a low-intensity activity of daily living rather than post-exercise recovery typically assessed during cardiopulmonary exercise testing.

Manuscript change
The definition of HRR has been clarified in the Methods section.

Comment 4
Please justify the classification of HF into new-onset and recurrent groups.

Response
We appreciate this important comment. In this study, heart failure groups were classified according to clinical course (new-onset versus recurrent HF) based on prior HF hospitalization history, because the primary aim was to examine physiological recovery dynamics during dressing as an activity of daily living rather than to differentiate pathophysiological HF subtypes. We have clarified this rationale in the revised manuscript. Moreover, we acknowledge that this classification may include heterogeneous HF phenotypes within each group, and this limitation has been explicitly discussed.

Manuscript change
The rationale for the classification has been clarified in the Methods and Discussion sections, and potential heterogeneity has been acknowledged in the Limitations section.

Comment 5
Medication effects (e.g., beta-blockers) may influence the results.

Response
Thank you for raising this important point. We agree that medications such as beta-blockers and other cardiovascular agents may influence heart rate and autonomic responses. Unfortunately, detailed medication timing data suitable for structured analysis were not available in the dataset. We have therefore acknowledged this issue as a study limitation and clarified that medication effects may have influenced the physiological responses observed.

Manuscript change
The potential influence of medication use has been described in the Limitations section.

Comment 6
Please ensure compliance with the PLOS data sharing policy.

Response
We appreciate this comment. The Data Availability Statement has been revised to ensure compliance with the PLOS ONE data sharing policy while protecting participant confidentiality. Specifically, we have clarified the ethical restrictions governing data sharing and provided a non-author institutional contact for data access requests.

Manuscript change
The Data Availability Statement has been revised accordingly.

Comment 7
ULSC interpretation suggests compensatory movement.

Response
Thank you for this insightful comment. We agree that the interpretation of ULSC required clarification. In the revised manuscript, we have clarified that ULSC should be interpreted primarily as an indicator of upper-limb activity volume rather than a direct marker of compensatory movement strategies. Because time-normalized ULSC did not differ significantly between groups, the results do not support clear evidence of compensatory upper-limb behavior.

Manuscript change
The interpretation of ULSC has been revised in the Discussion section.

**Reviewer 2**

Comment 1
Classification based solely on hospitalization history may not fully capture HF heterogeneity.

Response
Thank you for this thoughtful comment. We agree that heart failure is a heterogeneous syndrome and that classification based solely on hospitalization history may not fully capture important clinical and pathophysiological differences among patients. Nonetheless, the grouping strategy was based on clinical course because the primary aim was to examine recovery dynamics during an activity of daily living. We have clarified that the lack of additional stratifiers such as NYHA class, LVEF, and BNP/NT-proBNP may have contributed to heterogeneity within groups.

Manuscript change
This limitation has been clarified in the Limitations section.

Comment 2
Medication data such as ACEi/ARB/ARNI, beta-blockers, and MRA should be reported.

Response

Thank you for this important comment. We agree that medication use, including ACE inhibitors, angiotensin receptor blockers, angiotensin receptor–neprilysin inhibitors, beta-blockers, and mineralocorticoid receptor antagonists, may influence cardiovascular and autonomic responses. Nonetheless, detailed medication data suitable for structured analysis were not available in the present dataset. Medication regimens were managed individually as part of routine clinical care and were not collected in a standardized format for research analysis in this study. Therefore, we could not perform a systematic analysis of medication effects. In response to the reviewer’s comment, we have acknowledged this issue as a limitation of the study and clarified that medication use may have influenced the physiological responses observed.

Manuscript change

The absence of detailed medication data and the potential influence of medication use have been described in the Limitations section.

The lack of medication data has been described in the Limitations section.

Comment 3
HRV interpretation should be more cautious.

Response
We thank the reviewer for this important methodological comment. In response, we revised the Discussion section to clarify that HRV indices, such as LF/HF and LF-NU, should not be interpreted as simple indicators of sympathovagal balance, particularly under short-term, nonstationary conditions such as those in this study.

Manuscript change
The methodological limitations of HRV interpretation have been clarified in the Discussion section.

Comment 4
Temporal differences should not be overinterpreted.

Response
We appreciate this comment and agree with the reviewer’s concern. The manuscript has been revised to ensure that temporal patterns are described as descriptive observations rather than statistically confirmed group-specific responses. This revision was applied throughout the Abstract, Results, Discussion, and Limitations sections.

Manuscript change
Relevant sections of the manuscript have been revised to ensure cautious interpretation of temporal patterns.
