# Supplementary material for: Dressing-induced hemodynamic instability in patients with heart failure: Implications for nursing care
Source: PLoS One. 2026 Jun 11;21(6):e0351501. doi: 10.1371/journal.pone.0351501 (PMC13257999; doi:10.1371/journal.pone.0351501)
Supplement: Attachment — Submitted filename: Response_to_Reviewers_auresp_2.docx [file pone.0351501.s003.docx]

Response to Reviewers

Dear Academic Editor and Reviewers:

We sincerely thank the Academic Editor and reviewers for their careful evaluation of our revised manuscript entitled “Dressing-induced hemodynamic instability in patients with heart failure: Implications for nursing care” (PONE-D-25-63210R1). We are grateful that Reviewer #1 considered the manuscript suitable for acceptance. We also appreciate Reviewer #2’s constructive and insightful comments, which helped us improve the conceptual clarity, clinical characterization, and interpretive caution of the manuscript.

In response to Reviewer #2’s concerns, we have substantially revised the manuscript by clarifying the rationale for the grouping strategy, adding LVEF-based HF phenotype classification to Table 1, strengthening the limitations regarding BNP/NT-proBNP and medication data, and moderating interpretations where no significant group × time interactions were observed. The responses to all comments have been prepared and given below.

All changes are highlighted in red in the revised manuscript.

Reviewer #1

Comment:

The authors have revised the manuscript carefully and adequately addressed the points raised in the previous review. The revised version is clearer and more complete, and I have no further substantive concerns regarding the manuscript. I therefore consider the manuscript suitable for acceptance in its present form.

Response:

We sincerely thank Reviewer #1 for the positive evaluation and for recognizing the improvements made in the revised manuscript. We greatly appreciate the reviewer’s constructive comments throughout the review process.

Reviewer #2

General response:

We sincerely thank Reviewer #2 for the careful re-evaluation of our manuscript and for raising important concerns regarding the grouping strategy, clinical characterization, medication data, and interpretation of group-specific temporal patterns. We agree that these points are critical for improving the clinical interpretability and scientific rigor of the manuscript. Accordingly, we revised the manuscript to clarify that the present study was designed to examine recovery dynamics during activities of daily living according to the clinical course of heart failure (HF), rather than to compare ejection fraction (EF)-based physiological HF subtypes. In addition, we added left ventricular ejection fraction (LVEF)-based HF phenotype classifications and strengthened the limitations concerning unavailable B-type natriuretic peptide (BNP)/N-terminal pro-B-type natriuretic peptide (NT-proBNP) and medication data. Furthermore, we revised the Abstract, Results, Discussion, Limitations, and Conclusions to ensure that descriptive temporal patterns are interpreted cautiously in light of the absence of significant group × time interactions.

Comment 1. Misalignment between study objective and grouping strategy

Reviewer’s comment:

The authors state that the primary aim is to examine recovery dynamics during activities of daily living. However, patients are stratified based on hospitalization history (NO-HF vs. R-HF), which is an outcome-oriented variable rather than a physiological determinant. Recovery dynamics are more directly influenced by cardiac function, autonomic regulation, and disease severity. Therefore, the current grouping strategy appears conceptually misaligned with the study objective and its physiological focus. The appropriateness of this classification should be reconsidered.

Response:

We thank the reviewer for this important and insightful comment. We agree that hospitalization history is not a direct physiological determinant in the same way as cardiac function, autonomic regulation, or disease severity. Our intention was not to define physiological HF subtypes based on hospitalization history. Rather, we used hospitalization history as a clinically relevant indicator of disease trajectory, cumulative disease burden, and recurrent decompensation among clinically stable outpatients with HF.

To clarify this point, we have revised the manuscript to explicitly state that the grouping strategy was intended to reflect clinical course rather than EF-based physiological subtypes. In addition, we have clarified that the primary objective of this study was to characterize recovery dynamics during activities of daily living, not to compare EF-based pathophysiological phenotypes.

Changes made:

In the Materials and methods section, we have added/clarified the following text:

“This grouping strategy was selected to reflect cumulative disease burden and recurrent decompensation in clinically stable outpatients with HF, rather than to define physiological HF subtypes. As the primary objective of this study was to characterize recovery dynamics during activities of daily living rather than to compare ejection fraction-based pathophysiological phenotypes, hospitalization frequency was used as a clinically meaningful indicator of disease trajectory and readmission risk in patients with HF.” (lines 105–111)

In the Discussion section, we have further clarified:

“In the present study, HF groups were classified according to clinical course (new-onset vs. recurrent HF) to reflect cumulative disease burden and recurrent decompensation in daily clinical practice. Accordingly, the primary aim was to characterize recovery dynamics during activities of daily living rather than to compare ejection fraction-based pathophysiological HF phenotypes.” (lines 440–444)

We believe that these revisions address the reviewer’s concern by clarifying the conceptual basis of the grouping strategy and aligning it more explicitly with the study objective.

Comment 2. Insufficient characterization of HF phenotype and severity

Reviewer’s comment:

Key clinical variables essential for interpreting HF physiology are not reported, including LVEF-based phenotypes (HFpEF/HFrEF/HFmrEF) and biomarkers such as BNP or NT-proBNP. Without these data, it is difficult to interpret the hemodynamic and autonomic findings or to compare the groups appropriately. This limitation significantly weakens the clinical interpretability of the findings.

Response:

We thank the reviewer for this important comment. We agree that HF phenotype and severity markers are essential for interpreting hemodynamic and autonomic responses in patients with HF. In response, we have added LVEF-based HF phenotype classifications, including HFrEF, HFmrEF, and HFpEF, in Table 1,. This addition provides more detailed clinical characterization of the HF population and helps clarify the distribution of EF-based phenotypes across the NO-HF and R-HF groups.

However, we acknowledge that BNP and NT-proBNP data were not collected in the present study. We agree that the absence of these biomarkers limits detailed characterization of HF severity and physiological heterogeneity. Therefore, we have strengthened the Limitations section to explicitly state this issue and to caution readers regarding interpretation.

Changes made:

In the Results section, we have added:

“To improve clinical characterization of the HF population, LVEF-based HF phenotypes (HF with reduced ejection fraction, HF with mildly reduced ejection fraction, and HF with preserved ejection fraction) are additionally summarized in Table 1.” (lines 250–252)

In Table 1, we have added LVEF-based HF phenotype classifications:

HFrEF (<40%)

HFmrEF (40–49%)

HFpEF (≥50%)

We have also added the following footnote to Table 1:

“LVEF values are shown for patients with HF only. HF phenotypes were classified according to LVEF as follows: HFrEF, <40%; HFmrEF, 40–49%; and HFpEF, ≥50%.” (lines 256–258)

In the Discussion, we have added:

“Notably, LVEF-based HF phenotypes were distributed across both HF groups, suggesting that the observed recovery patterns were not attributable to a single EF category alone.” (lines 445–447)

In the Limitations section, we have revised the text to state:

“Although LVEF-based HF phenotypes were additionally summarized, biomarkers reflecting HF severity, including B-type natriuretic peptide and N-terminal pro-B-type natriuretic peptide, were not collected. Therefore, detailed characterization of disease severity and physiological heterogeneity across HF groups remains limited.” (lines 506–510)

We believe that these revisions improve the clinical characterization of the study population while transparently acknowledging the remaining limitation regarding HF severity biomarkers.

Comment 3. Lack of medication data

Reviewer’s comment:

The absence of information on guideline-directed medical therapy (e.g., beta-blockers, ACEi/ARB/ARNI, MRAs) represents a critical limitation. These medications have substantial effects on heart rate dynamics, blood pressure, and autonomic function. Without accounting for these factors, the interpretation of key outcomes such as HR, HRR, and SBP responses is highly uncertain.

Response:

We fully agree with the reviewer that guideline-directed medical therapy may substantially influence HR, HRR, SBP, and autonomic responses. In particular, beta-blockers and other cardiovascular medications may affect chronotropic responses, blood pressure regulation, and autonomic indices. Unfortunately, detailed medication data were not systematically collected in this study; therefore, we were unable to adjust for these factors statistically.

In response to this comment, we have strengthened the Limitations section to clearly state that detailed medication data, including beta-blockers, ACE inhibitors/ARBs/ARNIs, mineralocorticoid receptor antagonists, and diuretics, were not systematically collected. In addition, we have added cautionary language in the Discussion to avoid overinterpreting HR and HRR findings in the absence of medication information.

Changes made:

In the Discussion, we have added:

“Nonetheless, because medication use and clinical severity markers were unavailable in this study, this interpretation should be regarded as exploratory. Particularly, the potential influence of pharmacological HR modulation cannot be excluded.” (lines 457–460)

In the Limitations section, we have replaced the previous wording with:

“Fourth, detailed medication data, including β-blockers, angiotensin-converting enzyme (ACE) inhibitors/angiotensin II receptor blockers/angiotensin receptor–neprilysin inhibitors, mineralocorticoid receptor antagonists, and diuretics, were not systematically collected. These therapies may have influenced HR, HRR, SBP, and autonomic responses; therefore, the findings should be interpreted cautiously.” (lines 513–518)

We believe that these changes directly address the reviewer’s concern by explicitly acknowledging the absence of medication data and its potential influence on the key outcomes.

Comment 4. Inconsistency between statistical results and interpretation

Reviewer’s comment:

The manuscript reports no significant group × time interactions. Despite this, the discussion interprets group-specific temporal patterns (e.g., delayed peak responses in R-HF, biphasic SBP changes), which are not statistically supported. This may lead to overinterpretation of the data. The authors should moderate these interpretations and align them with the statistical results.

Response:

We thank the reviewer for this important comment. We agree that temporal patterns should not be interpreted as statistically confirmed group-specific trajectories in the absence of significant group × time interactions. In response, we have revised the Abstract, Results, Discussion, Limitations, and Conclusions sections to moderate the interpretation of temporal findings. Specifically, we have changed stronger expressions to more cautious wording, such as “descriptively,” “tended to,” “exploratory,” and “should be interpreted cautiously.”

We have also revised the descriptions of delayed HR peaks and SBP re-rise to clarify that these were descriptive observations rather than statistically confirmed interaction effects.

Changes made:

In the Abstract, we have revised the statement regarding the delayed HR peak:

“…R-HF descriptively showed a delayed peak around 5 min.” (lines 41–42)

In the Results section for SBP, we have revised the interpretation as follows:

“Subsequently, SBP in HCs promptly returned to baseline, whereas NO-HF and R-HF descriptively exhibited a small secondary SBP rise at approximately 15 min; however, this pattern should be interpreted cautiously because no significant group × time interaction was identified.” (lines 363–366)

In the Discussion, we have retained the clinical relevance of HRR but added caution:

“Nevertheless, because group × time interaction effects were not statistically significant, these findings should be interpreted cautiously and considered exploratory observations warranting confirmation in larger cohorts.” (lines 437–439)

We have also revised the interpretation of HF response patterns to avoid overstatement:

“These findings may suggest two complementary recovery response patterns relevant to bedside care.” (lines 487–488)

In the Limitations section, we have emphasized:

“For interpretation, SBP re-rise and the R-HF ~5-min HR peak are descriptive (exploratory) features; these patterns should therefore be interpreted cautiously because group × time interaction effects were not statistically significant.” (lines 524–526)

In the Conclusions, we have revised the interpretation as follows:

“Even a low-intensity dressing task revealed exploratory differences in recovery quality among groups: HRR ranked as R-HF > NO-HF > HC. NO-HF tended to show persistently higher HR levels with higher Borg ratings, while R-HF descriptively showed a blunted HR rise with delayed HR recovery. In addition, NO-HF and R-HF descriptively demonstrated a small SBP re-rise at approximately 15 min of recovery. Given that no significant group × time interactions were identified, these findings should be cautiously interpreted as exploratory recovery patterns requiring confirmation in larger studies.” (lines 534–540)

We believe that these revisions align the interpretation more closely with the statistical results and reduce the risk of overinterpretation.

We are grateful to Reviewer #2 for identifying these important issues.

We believe that these revisions have strengthened the manuscript and improved its clarity, rigor, and clinical interpretability. We sincerely thank the Academic Editor and reviewers for their valuable guidance and consideration.

Sincerely,

Sanae Oriyama

On behalf of all authors
