# Supplementary material for: Dressing-induced hemodynamic instability in patients with heart failure: Implications for nursing care
Source: PLoS One. 2026 Jun 11;21(6):e0351501. doi: 10.1371/journal.pone.0351501 (PMC13257999; doi:10.1371/journal.pone.0351501)
Supplement: Peer review history

# Peer review: Decision Letter 0

**DOI:** 10.1371/journal.pone.0351501.r001

**Authors:** Satoshi Higuchi

5 Apr 2026

PONE-D-25-63210

Dressing-induced hemodynamic instability in patients with heart failure: Implications for nursing care

PLOS One

Dear Dr. Oriyama,

Thank you for submitting your manuscript to PLOS ONE. After careful consideration, we feel that it has merit but does not fully meet PLOS ONE’s publication criteria as it currently stands. Therefore, we invite you to submit a revised version of the manuscript that addresses the points raised during the review process.

Please submit your revised manuscript by May 20 2026 11:59PM. If you will need more time than this to complete your revisions, please reply to this message or contact the journal office at plosone@plos.org. When you're ready to submit your revision, log on to https://www.editorialmanager.com/pone/ and select the 'Submissions Needing Revision' folder to locate your manuscript file.

Please include the following items when submitting your revised manuscript:

- A letter that responds to each point raised by the academic editor and reviewer(s). You should upload this letter as a separate file labeled 'Response to Reviewers'.
- A marked-up copy of your manuscript that highlights changes made to the original version. You should upload this as a separate file labeled 'Revised Manuscript with Track Changes'.
- An unmarked version of your revised paper without tracked changes. You should upload this as a separate file labeled 'Manuscript'.

If you would like to make changes to your financial disclosure, please include your updated statement in your cover letter. Guidelines for resubmitting your figure files are available below the reviewer comments at the end of this letter.

If applicable, we recommend that you deposit your laboratory protocols in protocols.io to enhance the reproducibility of your results. Protocols.io assigns your protocol its own identifier (DOI) so that it can be cited independently in the future. For instructions see: https://journals.plos.org/plosone/s/submission-guidelines#loc-laboratory-protocols. Additionally, PLOS ONE offers an option for publishing peer-reviewed Lab Protocol articles, which describe protocols hosted on protocols.io. Read more information on sharing protocols at https://plos.org/protocols?utm_medium=editorial-email&utm_source=authorletters&utm_campaign=protocols.

As the corresponding author, your ORCID iD is verified in the submission system and will appear in the published article. PLOS supports the use of ORCID, and we encourage all coauthors to register for an ORCID iD and use it as well. Please encourage your coauthors to verify their ORCID iD within the submission system before final acceptance, as unverified ORCID iDs will not appear in the published article. *Only* the individual author can complete the verification step; PLOS staff *cannot* verify ORCID iDs on behalf of authors.

We look forward to receiving your revised manuscript.

Kind regards,

Satoshi Higuchi

Academic Editor

PLOS One

Journal Requirements:

When submitting your revision, we need you to address these additional requirements.

1. Please ensure that your manuscript meets PLOS ONE's style requirements, including those for file naming. The PLOS ONE style templates can be found at

https://journals.plos.org/plosone/s/file?id=wjVg/PLOSOne_formatting_sample_main_body.pdf and

https://journals.plos.org/plosone/s/file?id=ba62/PLOSOne_formatting_sample_title_authors_affiliations.pdf.

2. Thank you for stating in your Funding Statement:

“This study was supported by the Japan Society for the Promotion of Science (JSPS), KAKENHI (grant number 23K09973 to TW). The funders played no role in the study design, data collection and analysis, decision to publish, or manuscript preparation.”

Please provide an amended statement that declares *all* the funding or sources of support (whether external or internal to your organization) received during this study, as detailed online in our guide for authors at http://journals.plos.org/plosone/s/submit-now. Please also include the statement “There was no additional external funding received for this study.” in your updated Funding Statement.

Please include your amended Funding Statement within your cover letter. We will change the online submission form on your behalf.

3. We note that you have indicated that there are restrictions to data sharing for this study. For studies involving human research participant data or other sensitive data, we encourage authors to share de-identified or anonymized data. However, when data cannot be publicly shared for ethical reasons, we allow authors to make their data sets available upon request. For information on unacceptable data access restrictions, please see http://journals.plos.org/plosone/s/data-availability#loc-unacceptable-data-access-restrictions.

Before we proceed with your manuscript, please address the following prompts:

a) If there are ethical or legal restrictions on sharing a de-identified data set, please explain them in detail (e.g., data contain potentially identifying or sensitive patient information, data are owned by a third-party organization, etc.) and who has imposed them (e.g., a Research Ethics Committee or Institutional Review Board, etc.). Please also provide contact information for a data access committee, ethics committee, or other institutional body to which data requests may be sent.

b) If there are no restrictions, please upload the minimal anonymized data set necessary to replicate your study findings to a stable, public repository and provide us with the relevant URLs, DOIs, or accession numbers. Please see http://www.bmj.com/content/340/bmj.c181.long for guidelines on how to de-identify and prepare clinical data for publication. For a list of recommended repositories, please see https://journals.plos.org/plosone/s/recommended-repositories. You also have the option of uploading the data as Supporting Information files, but we would recommend depositing data directly to a data repository if possible.

Please update your Data Availability statement in the submission form accordingly.

4. In this instance it seems there may be acceptable restrictions in place that prevent the public sharing of your minimal data. However, in line with our goal of ensuring long-term data availability to all interested researchers, PLOS’ Data Policy states that authors cannot be the sole named individuals responsible for ensuring data access (http://journals.plos.org/plosone/s/data-availability#loc-acceptable-data-sharing-methods).

Data requests to a non-author institutional point of contact, such as a data access or ethics committee, helps guarantee long term stability and availability of data. Providing interested researchers with a durable point of contact ensures data will be accessible even if an author changes email addresses, institutions, or becomes unavailable to answer requests.

Before we proceed with your manuscript, please also provide non-author contact information (phone/email/hyperlink) for a data access committee, ethics committee, or other institutional body to which data requests may be sent. If no institutional body is available to respond to requests for your minimal data, please consider if there any institutional representatives who did not collaborate in the study, and are not listed as authors on the manuscript, who would be able to hold the data and respond to external requests for data access? If so, please provide their contact information (i.e., email address). Please also provide details on how you will ensure persistent or long-term data storage and availability.

5. If the reviewer comments include a recommendation to cite specific previously published works, please review and evaluate these publications to determine whether they are relevant and should be cited. There is no requirement to cite these works unless the editor has indicated otherwise.

Additional Editor Comments (if provided):

We apologize for the delay in completing the review and appreciate your patience.

[Note: HTML markup is below. Please do not edit.]

Reviewers' comments:

Reviewer's Responses to Questions

**Comments to the Author**

1. Is the manuscript technically sound, and do the data support the conclusions?

The manuscript must describe a technically sound piece of scientific research with data that supports the conclusions. Experiments must have been conducted rigorously, with appropriate controls, replication, and sample sizes. The conclusions must be drawn appropriately based on the data presented.

Reviewer #1: Yes

Reviewer #2: Partly

**********

2. Has the statistical analysis been performed appropriately and rigorously?

Reviewer #1: Yes

Reviewer #2: Yes

**********

3. Have the authors made all data underlying the findings in their manuscript fully available?

The PLOS Data policy requires authors to make all data underlying the findings described in their manuscript fully available without restriction, with rare exception (please refer to the Data Availability Statement in the manuscript PDF file). The data should be provided as part of the manuscript or its supporting information, or deposited to a public repository. For example, in addition to summary statistics, the data points behind means, medians and variance measures should be available. If there are restrictions on publicly sharing data—e.g. participant privacy or use of data from a third party—those must be specified.

Reviewer #1: Yes

Reviewer #2: Yes

**********

4. Is the manuscript presented in an intelligible fashion and written in standard English?

PLOS ONE does not copyedit accepted manuscripts, so the language in submitted articles must be clear, correct, and unambiguous. Any typographical or grammatical errors should be corrected at revision, so please note any specific errors here.

Reviewer #1: Yes

Reviewer #2: Yes

**********

5. Review Comments to the Author

Please use the space provided to explain your answers to the questions above. You may also include additional comments for the author, including concerns about dual publication, research ethics, or publication ethics. (Please upload your review as an attachment if it exceeds 20,000 characters)

Reviewer #1: Thank you for the opportunity to review this interesting manuscript. The study addresses an underexplored but clinically relevant topic—physiological responses during dressing in patients with heart failure (HF). The integration of hemodynamic, autonomic, subjective, and behavioral measures is commendable. However, several methodological and interpretative aspects require clarification or further justification to strengthen the manuscript. My specific comments and questions are outlined below.

1). The sample size is relatively small, and no significant group × time interactions were observed; how do the authors justify that the study is sufficiently powered to support the conclusions, particularly regarding temporal differences between groups?

2). Given that the main hypothesis involves differences in recovery patterns, how do the authors interpret their findings in light of the absence of significant interaction effects?

3). The definition of heart rate recovery (HRR) used in this study differs from commonly used clinical indices; what is the rationale for this definition, and how does it compare with established HRR measures?

4). The classification of heart failure into de novo and recurrent groups is based on hospitalization history; what is the justification for this approach, and how do the authors account for potential heterogeneity within these groups?

5). Considering that medications such as beta-blockers can significantly affect heart rate and autonomic responses, how were medication effects controlled for or accounted for in the analysis?

6). The data availability statement indicates that the data cannot be shared publicly due to identifiable information; how do the authors plan to ensure compliance with PLOS ONE’s data sharing policy?

7). Upper-limb sensor count (ULSC) showed no significant differences between groups after adjustment, yet is discussed as reflecting compensatory behavior; how do the authors reconcile this inconsistency in interpretation?

Reviewer #2: I have read the article by Sanae Oriyama, et al entitled " Dressing-induced hemodynamic instability in patients with heart failure: Implications for

nursing care”.

In this manuscript, the authors investigated the simultaneous hemodynamic, autonomic, subjective symptom burden, and behavioral responses to a standardized dressing task in HF patients, compared with healthy controls.

The manuscript is interesting and the results are clinically interpretable. The authors also appropriately acknowledge the study limitations. However, several concerns should be addressed before the manuscript can be considered for publication.

1. While the NO-HF vs. R-HF classification is clinically intuitive, grouping patients solely based on hospitalization frequency may not fully capture the heterogeneity of HF. Key variables such as NYHA class, LVEF, and BNP appear to play a secondary role in the current framework. In particular, differences between HFpEF and HFrEF, which are known to have distinct pathophysiological and clinical characteristics, are not explicitly accounted for. The author should provide the data regarding HFrEF/HFmrEF/HFpEF and BNP or NT-proBNP level as the baseline characteristics in Table 1. Furthermore, addressing or discussing this point in more detail may help to further strengthen the interpretation of the results.

2. The author should also provide the data of medications, such as ACEi/ARB/ARNIs, beta-blockers, and MRAs.

3. The interpretation of HRV findings may benefit from a more cautious and consistent framework. While some indices (e.g., CvRR and LF) showed group differences, other key parameters such as HF, LF/HF, and group × time interactions were not significant. Given the ongoing methodological concerns regarding the physiological interpretation of LF and LF/HF, particularly under short-term and non-stationary conditions, the current findings may not fully support definitive conclusions regarding autonomic balance or differential autonomic regulation between groups. Clarifying these limitations and avoiding overinterpretation of selected HRV indices would strengthen the manuscript.

4. The manuscript reports that no significant group × time interactions were identified. However, in several instances, the subsequent descriptions appear to interpret temporal differences between groups, such as differences in peak timing (e.g., “R-HF peaked at 5 min”) or recovery patterns. The current description may lead to overinterpretation of between-group differences in temporal patterns. The author should use more cautious wording used to describe group-specific temporal responses.

**********

6. PLOS authors have the option to publish the peer review history of their article (what does this mean?). If published, this will include your full peer review and any attached files.

If you choose “no”, your identity will remain anonymous but your review may still be made public.

**Do you want your identity to be public for this peer review?** For information about this choice, including consent withdrawal, please see our Privacy Policy.

Reviewer #1: No

Reviewer #2: No

**********

[NOTE: If reviewer comments were submitted as an attachment file, they will be attached to this email and accessible via the submission site. Please log into your account, locate the manuscript record, and check for the action link "View Attachments". If this link does not appear, there are no attachment files.]

To ensure your figures meet our technical requirements, please review our figure guidelines: https://journals.plos.org/plosone/s/figures

You may also use PLOS’s free figure tool, NAAS, to help you prepare publication quality figures: https://journals.plos.org/plosone/s/figures#loc-tools-for-figure-preparation.

NAAS will assess whether your figures meet our technical requirements by comparing each figure against our figure specifications.

# Author comment: Author response to Decision Letter 1

**DOI:** 10.1371/journal.pone.0351501.r002

23 Apr 2026

Response to Reviewers document is uploaded in Attach Files section for review.

Attachment Submitted filename: Response to reviewers.docx

# Peer review: Decision Letter 1

**DOI:** 10.1371/journal.pone.0351501.r003

**Authors:** Satoshi Higuchi

6 May 2026

PONE-D-25-63210R1

Dressing-induced hemodynamic instability in patients with heart failure: Implications for nursing care

PLOS One

Dear Dr. Oriyama,

Thank you for submitting your manuscript to PLOS ONE. After careful consideration, we feel that it has merit but does not fully meet PLOS ONE’s publication criteria as it currently stands. Therefore, we invite you to submit a revised version of the manuscript that addresses the points raised during the review process.

Please submit your revised manuscript by Jun 20 2026 11:59PM. If you will need more time than this to complete your revisions, please reply to this message or contact the journal office at plosone@plos.org. When you're ready to submit your revision, log on to https://www.editorialmanager.com/pone/ and select the 'Submissions Needing Revision' folder to locate your manuscript file.

Please include the following items when submitting your revised manuscript:

- A letter that responds to each point raised by the academic editor and reviewer(s). You should upload this letter as a separate file labeled 'Response to Reviewers'.
- A marked-up copy of your manuscript that highlights changes made to the original version. You should upload this as a separate file labeled 'Revised Manuscript with Track Changes'.
- An unmarked version of your revised paper without tracked changes. You should upload this as a separate file labeled 'Manuscript'.

If you would like to make changes to your financial disclosure, please include your updated statement in your cover letter. Guidelines for resubmitting your figure files are available below the reviewer comments at the end of this letter.

If applicable, we recommend that you deposit your laboratory protocols in protocols.io to enhance the reproducibility of your results. Protocols.io assigns your protocol its own identifier (DOI) so that it can be cited independently in the future. For instructions see: https://journals.plos.org/plosone/s/submission-guidelines#loc-laboratory-protocols. Additionally, PLOS ONE offers an option for publishing peer-reviewed Lab Protocol articles, which describe protocols hosted on protocols.io. Read more information on sharing protocols at https://plos.org/protocols?utm_medium=editorial-email&utm_source=authorletters&utm_campaign=protocols.

As the corresponding author, your ORCID iD is verified in the submission system and will appear in the published article. PLOS supports the use of ORCID, and we encourage all coauthors to register for an ORCID iD and use it as well. Please encourage your coauthors to verify their ORCID iD within the submission system before final acceptance, as unverified ORCID iDs will not appear in the published article. *Only* the individual author can complete the verification step; PLOS staff *cannot* verify ORCID iDs on behalf of authors.

We look forward to receiving your revised manuscript.

Kind regards,

Satoshi Higuchi

Academic Editor

PLOS One

**Journal Requirements:**

If the reviewer comments include a recommendation to cite specific previously published works, please review and evaluate these publications to determine whether they are relevant and should be cited. There is no requirement to cite these works unless the editor has indicated otherwise.

[Note: HTML markup is below. Please do not edit.]

Reviewers' comments:

Reviewer's Responses to Questions

**Comments to the Author**

1. If the authors have adequately addressed your comments raised in a previous round of review and you feel that this manuscript is now acceptable for publication, you may indicate that here to bypass the “Comments to the Author” section, enter your conflict of interest statement in the “Confidential to Editor” section, and submit your "Accept" recommendation.

Reviewer #1: All comments have been addressed

Reviewer #2: (No Response)

**********

2. Is the manuscript technically sound, and do the data support the conclusions?

The manuscript must describe a technically sound piece of scientific research with data that supports the conclusions. Experiments must have been conducted rigorously, with appropriate controls, replication, and sample sizes. The conclusions must be drawn appropriately based on the data presented.

Reviewer #1: Yes

Reviewer #2: Partly

**********

3. Has the statistical analysis been performed appropriately and rigorously?

Reviewer #1: Yes

Reviewer #2: Yes

**********

4. Have the authors made all data underlying the findings in their manuscript fully available?

The PLOS Data policy requires authors to make all data underlying the findings described in their manuscript fully available without restriction, with rare exception (please refer to the Data Availability Statement in the manuscript PDF file). The data should be provided as part of the manuscript or its supporting information, or deposited to a public repository. For example, in addition to summary statistics, the data points behind means, medians and variance measures should be available. If there are restrictions on publicly sharing data—e.g. participant privacy or use of data from a third party—those must be specified.

Reviewer #1: Yes

Reviewer #2: Yes

**********

5. Is the manuscript presented in an intelligible fashion and written in standard English?

PLOS ONE does not copyedit accepted manuscripts, so the language in submitted articles must be clear, correct, and unambiguous. Any typographical or grammatical errors should be corrected at revision, so please note any specific errors here.

Reviewer #1: Yes

Reviewer #2: Yes

**********

6. Review Comments to the Author

Please use the space provided to explain your answers to the questions above. You may also include additional comments for the author, including concerns about dual publication, research ethics, or publication ethics. (Please upload your review as an attachment if it exceeds 20,000 characters)

**Reviewer #1:** The authors have revised the manuscript carefully and adequately addressed the points raised in the previous review. The revised version is clearer and more complete, and I have no further substantive concerns regarding the manuscript. I therefore consider the manuscript suitable for acceptance in its present form.

**Reviewer #2:** The authors addressed the reviewers' concerns. However, there are several residual concerns, which I would like to have addressed by the authors.

1. Misalignment between study objective and grouping strategy

The authors state that the primary aim is to examine recovery dynamics during activities of daily living. However, patients are stratified based on hospitalization history (NO-HF vs. R-HF), which is an outcome-oriented variable rather than a physiological determinant. Recovery dynamics are more directly influenced by cardiac function, autonomic regulation, and disease severity. Therefore, the current grouping strategy appears conceptually misaligned with the study objective and its physiological focus. The appropriateness of this classification should be reconsidered.

2. Insufficient characterization of HF phenotype and severity

Key clinical variables essential for interpreting HF physiology are not reported, including LVEF-based phenotypes (HFpEF/HFrEF/HFmrEF) and biomarkers such as BNP or NT-proBNP. Without these data, it is difficult to interpret the hemodynamic and autonomic findings or to compare the groups appropriately. This limitation significantly weakens the clinical interpretability of the findings.

3. Lack of medication data

The absence of information on guideline-directed medical therapy (e.g., beta-blockers, ACEi/ARB/ARNI, MRAs) represents a critical limitation. These medications have substantial effects on heart rate dynamics, blood pressure, and autonomic function. Without accounting for these factors, the interpretation of key outcomes such as HR, HRR, and SBP responses is highly uncertain.

4. Inconsistency between statistical results and interpretation

The manuscript reports no significant group × time interactions. Despite this, the discussion interprets group-specific temporal patterns (e.g., delayed peak responses in R-HF, biphasic SBP changes), which are not statistically supported. This may lead to overinterpretation of the data. The authors should moderate these interpretations and align them with the statistical results.

Taken together, these issues raise concerns regarding both the conceptual design and the interpretability of the results. Substantial revision, including improved clinical characterization and more cautious interpretation, would be necessary to support the conclusions.

**********

7. PLOS authors have the option to publish the peer review history of their article (what does this mean?). If published, this will include your full peer review and any attached files.

If you choose “no”, your identity will remain anonymous but your review may still be made public.

**Do you want your identity to be public for this peer review?** For information about this choice, including consent withdrawal, please see our Privacy Policy.

Reviewer #1: No

Reviewer #2: No

**********

[NOTE: If reviewer comments were submitted as an attachment file, they will be attached to this email and accessible via the submission site. Please log into your account, locate the manuscript record, and check for the action link "View Attachments". If this link does not appear, there are no attachment files.]

To ensure your figures meet our technical requirements, please review our figure guidelines: https://journals.plos.org/plosone/s/figures

You may also use PLOS’s free figure tool, NAAS, to help you prepare publication quality figures: https://journals.plos.org/plosone/s/figures#loc-tools-for-figure-preparation.

NAAS will assess whether your figures meet our technical requirements by comparing each figure against our figure specifications.

# Author comment: Author response to Decision Letter 2

**DOI:** 10.1371/journal.pone.0351501.r004

24 May 2026

Attached

Attachment Submitted filename: Response_to_Reviewers_auresp_2.docx

# Peer review: Decision Letter 2

**DOI:** 10.1371/journal.pone.0351501.r005

**Authors:** Satoshi Higuchi

29 May 2026

Dressing-induced hemodynamic instability in patients with heart failure: Implications for nursing care

PONE-D-25-63210R2

Dear Dr. Oriyama,

We’re pleased to inform you that your manuscript has been judged scientifically suitable for publication and will be formally accepted for publication once it meets all outstanding technical requirements.

Within one week, you’ll receive an e-mail detailing the required amendments. When these have been addressed, you’ll receive a formal acceptance letter and your manuscript will be scheduled for publication.

An invoice will be generated when your article is formally accepted. Please note, if your institution has a publishing partnership with PLOS and your article meets the relevant criteria, all or part of your publication costs will be covered. Please make sure your user information is up-to-date by logging into Editorial Manager at Editorial Manager® and clicking the ‘Update My Information' link at the top of the page. For questions related to billing, please contact billing support.

If your institution or institutions have a press office, please notify them about your upcoming paper to help maximize its impact. If they’ll be preparing press materials, please inform our press team as soon as possible -- no later than 48 hours after receiving the formal acceptance. Your manuscript will remain under strict press embargo until 2 pm Eastern Time on the date of publication. For more information, please contact onepress@plos.org.

Kind regards,

Satoshi Higuchi

Academic Editor

PLOS One

Additional Editor Comments (optional):

Reviewers' comments:

Reviewer's Responses to Questions

**Comments to the Author**

1. If the authors have adequately addressed your comments raised in a previous round of review and you feel that this manuscript is now acceptable for publication, you may indicate that here to bypass the “Comments to the Author” section, enter your conflict of interest statement in the “Confidential to Editor” section, and submit your "Accept" recommendation.

Reviewer #2: All comments have been addressed

**********

2. Is the manuscript technically sound, and do the data support the conclusions?

The manuscript must describe a technically sound piece of scientific research with data that supports the conclusions. Experiments must have been conducted rigorously, with appropriate controls, replication, and sample sizes. The conclusions must be drawn appropriately based on the data presented.

Reviewer #2: Yes

**********

3. Has the statistical analysis been performed appropriately and rigorously?

Reviewer #2: Yes

**********

4. Have the authors made all data underlying the findings in their manuscript fully available?

The PLOS Data policy requires authors to make all data underlying the findings described in their manuscript fully available without restriction, with rare exception (please refer to the Data Availability Statement in the manuscript PDF file). The data should be provided as part of the manuscript or its supporting information, or deposited to a public repository. For example, in addition to summary statistics, the data points behind means, medians and variance measures should be available. If there are restrictions on publicly sharing data—e.g. participant privacy or use of data from a third party—those must be specified.

Reviewer #2: Yes

**********

5. Is the manuscript presented in an intelligible fashion and written in standard English?

PLOS ONE does not copyedit accepted manuscripts, so the language in submitted articles must be clear, correct, and unambiguous. Any typographical or grammatical errors should be corrected at revision, so please note any specific errors here.

Reviewer #2: Yes

**********

6. Review Comments to the Author

Please use the space provided to explain your answers to the questions above. You may also include additional comments for the author, including concerns about dual publication, research ethics, or publication ethics. (Please upload your review as an attachment if it exceeds 20,000 characters)

Reviewer #2: Thank you for your careful revisions and detailed responses to the reviewers’ comments. I believe

the manuscript has been substantially improved and is now acceptable for publication.

**********

7. PLOS authors have the option to publish the peer review history of their article (what does this mean?). If published, this will include your full peer review and any attached files.

If you choose “no”, your identity will remain anonymous but your review may still be made public.

**Do you want your identity to be public for this peer review?** For information about this choice, including consent withdrawal, please see our Privacy Policy.

Reviewer #2: No

**********

# Editor report: Acceptance letter

**DOI:** 10.1371/journal.pone.0351501.r006

**Authors:** Satoshi Higuchi

PONE-D-25-63210R2

PLOS One

Dear Dr. Oriyama,

I'm pleased to inform you that your manuscript has been deemed suitable for publication in PLOS One. Congratulations! Your manuscript is now being handed over to our production team.

At this stage, our production department will prepare your paper for publication. This includes ensuring the following:

* All references, tables, and figures are properly cited

* All relevant supporting information is included in the manuscript submission,

* There are no issues that prevent the paper from being properly typeset

You will receive further instructions from the production team, including instructions on how to review your proof when it is ready. Please keep in mind that we are working through a large volume of accepted articles, so please give us a few days to review your paper and let you know the next and final steps.

Lastly, if your institution or institutions have a press office, please let them know about your upcoming paper now to help maximize its impact. If they'll be preparing press materials, please inform our press team within the next 48 hours. Your manuscript will remain under strict press embargo until 2 pm Eastern Time on the date of publication. For more information, please contact onepress@plos.org.

You will receive an invoice from PLOS for your publication fee after your manuscript has reached the completed accept phase. If you receive an email requesting payment before acceptance or for any other service, this may be a phishing scheme. Learn how to identify phishing emails and protect your accounts at https://explore.plos.org/phishing.

If we can help with anything else, please email us at customercare@plos.org.

Thank you for submitting your work to PLOS ONE and supporting open access.

Kind regards,

PLOS ONE Editorial Office Staff

on behalf of

Dr. Satoshi Higuchi

Academic Editor

PLOS One
